# Design of a Broadband Array Pattern of Underwater Cymbal Transducers

**DOI:** 10.3390/s21186119

**Published:** 2021-09-12

**Authors:** Donghyun Kim, Hayeong Shim, Changmin Oh, Kyungseop Kim, Heeseon Seo, Yongrae Roh

**Affiliations:** 1 School of Mechanical Engineering, Kyungpook National University, Daegu 41566, Korea; roy4435@naver.com (D.K.); hiyo3@naver.com (H.S.); radicalohcm@gmail.com (C.O.); 2 Agency for Defense Development, Changwon 51678, Korea; seop@add.re.kr (K.K.); hsseo@add.re.kr (H.S.)

**Keywords:** cymbal transducer, array pattern, optimal design, broadband

## Abstract

Cymbal transducers are frequently used as an array rather than a single element because of their high quality factor and low energy conversion efficiency. When used as an array, cymbal transducers are likely to have a big change in their frequency characteristics due to the interaction with neighboring elements. In this study, we designed an array pattern of cymbal transducers to achieve a wide frequency bandwidth using this property. First, cymbal transducers with specific center frequencies were designed. Next, a 2 × 2 planar array was constructed with the designed transducers, where dielectric polarity directions of the transducers were divided into two cases (i.e., same and different). For the array, the effect of the difference in the center frequencies and the spacing between the transducers on the acoustic characteristics of the entire array was analyzed. Based on the results, the structural pattern of the array was optimized to have the maximum fractional bandwidth while maintaining the transmitting voltage response over a given requirement. The design validity was verified by making cymbal array prototypes, followed by measuring their performances and comparing them with that of the design.

## 1. Introduction

Many studies are being conducted to develop the technology for underwater sensor networks (UWSN) using multiple sensor nodes. A representative example of this technology is Seaweb, which consists of sensor and intermediate nodes and gateways [1]. Acoustic transducers used as the sensor nodes in a UWSN system are the key factors in determining the system performance [2]. For high-speed communication and large-capacity data transmission, in particular, an acoustic sensor with wideband frequency characteristics is essential [3].

This study investigates a cymbal transducer used as an underwater acoustic sensor that satisfies the abovementioned conditions. Developed by Newnham et al., this transducer is a miniaturized version of the Class V flextensional transducer [4]. It has a piezoceramic disk sandwiched between cymbal-shaped metal caps that acts as a mechanical transformer. In comparison with other types of underwater transducers, they are easy to manufacture and have advantages such as high power, small weight, and low resonant frequency considering their small volume [5]. In addition, the cymbal transducer can be easily integrated into various array forms due to its small size, which is suitable for large-area and restricted-volume arrays [6]. When cymbal transducers are used in an array, they cannot only achieve high power output, but also yield broadband characteristics caused by their interaction with the neighboring elements in the array [7]. However, cymbal transducers generally exhibit a high quality factor and a low energy conversion efficiency, which require a careful and accurate design of their array structure [8].

Many studies on the cymbal array have been conducted. For instance, Zhang et al. analyzed the cymbal array characteristics by combining the finite element method (FEM) with integral equations and compared the analysis results with the measured performance of a prototype array [9]. Tressler et al. designed an underwater projector by arranging cymbal transducers in series, parallel, or a combination of both [10]. Luis et al. designed and fabricated a 3 × 3 circular cymbal array and a 3 × 1 rectangular cymbal array [11]. Maione et al. compared the performance of a basic cymbal array composed of four parallel transducers with that of a stack array, in which piezoelectric discs are connected mechanically in series and electrically in parallel [12]. A comparative evaluation of receiving sensitivity and directivity characteristics of flat cymbal arrays for hydrophones was also conducted [13,14]. Some studies were conducted to widen the bandwidth. Tressler et al. showed that the bandwidth could be controlled by the metal cap material and the presence or absence of potting [15]. Meanwhile, Zhang et al. analyzed the difference in array performance according to the array interaction [6]. Newnham et al. compared the transmitting voltage response (TVR) by fabricating two types of arrays, i.e., 3 × 3 and 5 × 20 arrays, which showed that the bandwidth could become broader as the number of transducers increased [16].

The abovementioned studies focused on the development and characterization of an array consisting only of cymbal transducers with an identical structure and a uniform planar array pattern. These previous works have shown several structural parameters that can be utilized to control the cymbal array bandwidth, such as different center frequencies and dielectric polarity directions of the constituent cymbals, and the inter-element spacing between the cymbal transducers. These parameters have noticeable effects on the frequency characteristics of the array. Hence, the cymbal array bandwidth can be broadened through a systematic process to incorporate the effects of all parameters in the array structure design, which has not yet been conducted thus far.

Shim and Roh worked on the design of individual cymbal transducers [17]. In this study, we work on the design of the array pattern of cymbal transducers to achieve a broadband frequency response. For this purpose, we first analyze the effect of the structural parameters on the acoustic characteristics of the array that is composed of the cymbal transducers designed by the method in [18]. Based on the results, we design the cymbal array pattern to have the maximum fractional bandwidth (FBW) while satisfying a specific output performance through an optimization process. Subsequently, we fabricate experimental prototypes of the cymbal array to have the optimized array pattern and measure their performance. The validity of the new array pattern is verified by comparing the measured performance with the design.

## 2. Configuration of a 2 × 2 Cymbal Array

We selected a 2 × 2 array as a representative structure of a cymbal array. It is the simplest of all the possible array structures, but we expected that the effects of structural parameters observed in a 2 × 2 array would be the same as those in higher-dimension arrays, which we confirmed in our preliminary analysis. As a result, the analysis with a 2 × 2 array could be more time-saving and efficient.

**Figure 1:**
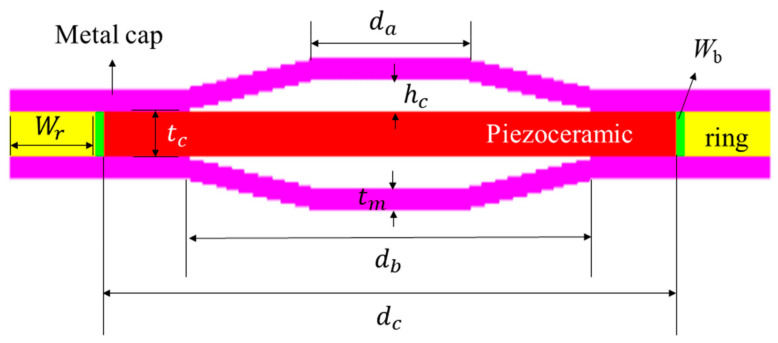
Schematic structure of the cymbal transducer.

Prior to designing a 2 × 2 array, we first constructed a finite element analysis (FEA) model of the cymbal transducer using a commercial FEA program, PZFlex^®^, and analyzed the model’s acoustic characteristics. Figure 1 depicts the FEA model showing the structure of a single cymbal transducer, where *d*_a_, *d*_b_, and *d*_c_ are the apex, base, and ceramic disk diameters, respectively, and *h*_c_, *t*_m_, *W*_r_, and *W*_b_ are the cavity height, cap thickness, ring width, and bond width, respectively. For the cymbal transducer structure, the metal caps were bonded to the top and bottom surfaces of the piezoceramic disk. The materials for the piezoceramic, metal cap, and ring were PZT-5A, brass, and polyether ether ketone, respectively. The material properties were taken from [18]. The thickness of the bonding layer was controlled to be less than 10 μm so that its effect could be negligible [19].

**Figure 2:**
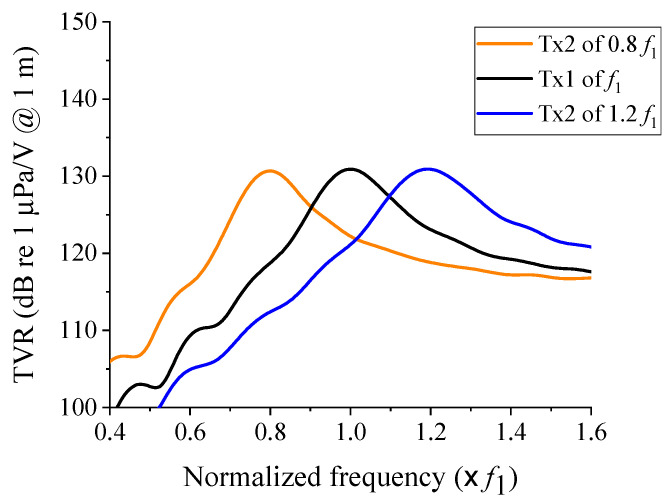
Underwater TVR spectra of the designed Tx_1_ and two Tx_2_ transducers.

The cymbal transducers constituting the 2 × 2 array were designed with specific center frequencies and TVR levels [17]. First, the structure of a cymbal transducer (Tx_1_) with a center frequency of *f*_1_ was designed. Table 1 presents Tx_1′_s dimensions. Tx_1_ was used as a reference transducer. Next, another cymbal transducer (Tx_2_) was designed to have *f*_2_ as the center frequency, to compose the array in combination with Tx_1_. *f*_2_ was set with values that were 20% higher or lower than *f*_1_. Figure 2 shows the TVR spectra of the designed Tx_1_ and two Tx_2_s. Table 2 presents a comparison of the quantitative acoustic characteristics of each transducer. All frequencies were normalized to the center frequency *f*_1_ of Tx_1_. The fractional bandwidth was calculated by dividing the −3 dB bandwidth with the center frequency of each transducer.

**Figure 3:**
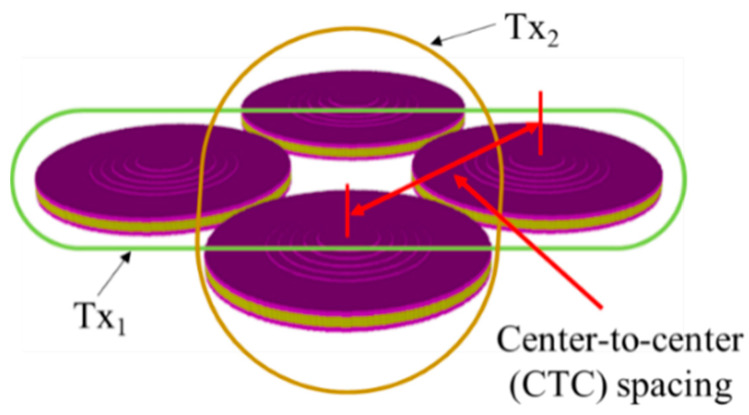
A 3D FEA model of the 2 × 2 cymbal array.

A planar arrangement was constructed (Figure 3) using the designed Tx_1_ and Tx_2_ transducers. The transducers that diagonally face each other were Tx_1_ and Tx_2_. The Tx_2_ pair can have a center frequency of either 0.8 *f*_1_ or 1.2 *f*_1_.

With PZFlex^®^, a three-dimensional FEA model of the array immersed in water was constructed to simulate the TVR. A sufficiently large amount of water was set outside the array to preserve the underwater transmission characteristics of the planar array. The array pattern had two orthogonal symmetric planes, so only a quarter model was analyzed to save calculation time. The element size in the FEA model was 0.15 mm. The whole model consisted of 127 million elements and 128 million nodes. Absorption boundary conditions were enforced onto the outer edges of the water domain such that sound reflection would not occur at the edges. The sound pressure emitted from the array was calculated at a far-field point in the vertical direction from the upper cap.

## 3. Effect of the Structural Parameters on the 2 × 2 Cymbal Array Performance

This study aimed to design an array pattern that can achieve the widest bandwidth. Accordingly, the influence of the structural parameters on the cymbal array performance was analyzed by using the FEM. The structural parameters considered herein were the dielectric polarity direction of the piezoceramic disks in Tx_1_ and Tx_2_, the center frequency of Tx_2_ relative to that of Tx_1_, and the spacing between the centers of Tx_1_ and Tx_2_.

We observed the effect of the dielectric polarity direction of a piezoceramic disk by analyzing cases where the PZT disks in Tx_1_ and Tx_2_ had the same polarity direction or opposite directions. To assess the effect of the difference in transducer center frequencies, the center frequency of Tx_1_ was fixed to the reference frequency *f*_1_, while that of Tx_2_ (*f*_2_) was changed to 0.8 *f*_1_, 1.0 *f*_1_, and 1.2 *f*_1_, in sequence. The fractional bandwidth of each cymbal transducer was less than 20%; hence, the amount of the center frequency change was set to a maximum of 20% of *f*_1_. Regarding the effect of the center-to-center (CTC) spacing, the spacing was changed from 0.3 λ to 0.36 λ and 0.42 λ, where λ is the wavelength of the sound wave in water at *f*_1_. The minimum CTC spacing was set to 0.3 λ considering the cymbal transducer radius. The spacing was increased by 20% intervals. Cymbal transducers are reciprocal transducers and their TVR spectra were analyzed to evaluate the performance [20].

**Figure 4:**
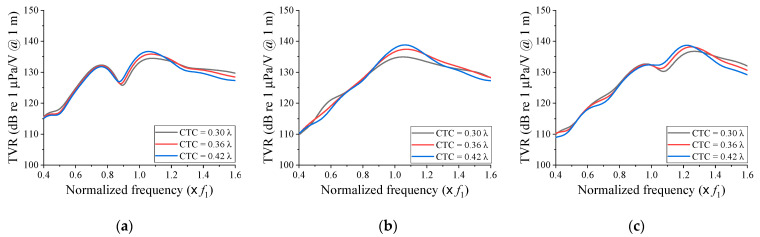
Comparison of the TVR spectra in relation to the center frequency difference and the CTC spacing when the polarity directions of Tx_1_ and Tx_2_ were the same: (**a**) *f*_2_ = 0.8 *f*_1_; (**b**) *f*_2_ = *f*_1_; (**c**) *f*_2_ = 1.2 *f*_1_.

Figure 4 shows the TVR spectra of the cymbal array according to the change of *f*_2_ and the CTC spacing when Tx_1_ and Tx_2_ had the same dielectric polarity direction. For all cases, the −3 dB bandwidth increased, while the maximum TVR decreased as the CTC spacing became shorter. The fractional bandwidth was the widest when the CTC spacing was 0.30 λ for all *f*_2_s. This was 29.9% when *f*_2_ = 0.8 *f*_1_, 37.1% when *f*_2_ = *f*_1_, and 26.4% when *f*_2_ = 1.2 *f*_1_. For all cases, the array bandwidth was significantly wider than that of the individual transducers illustrated in Figure 2. In Figure 4a,c, a notch was observed in the frequency range between *f*_1_ and *f*_2_, which was caused by the acoustic impedances of Tx_1_ and Tx_2_ being out of phase with each other in that range.

**Figure 5:**
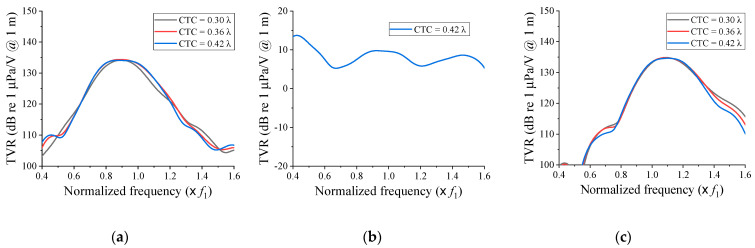
Comparison of the TVR spectra in relation to the center frequency difference and the CTC spacing when the polarity directions of Tx_1_ and Tx_2_ were opposite: (**a**) *f*_2_ = 0.8 *f*_1_; (**b**) *f*_2_ = *f*_1_; (**c**) *f*_2_ = 1.2 *f*_1_.

Figure 5 shows the variations of the TVR spectrum of the cymbal array according to the change of *f*_2_ and the CTC spacing when the dielectric polarity directions of Tx_1_ and Tx_2_ were opposite to each other. Contrary to that in Figure 4, the bandwidth increased, while the TVR level decreased as the CTC spacing became longer. In Figure 5a,c, the fractional bandwidth was the widest when the CTC spacing was 0.42 λ. This was 33.0% when *f*_2_ = 0.8 *f*_1_ and 25.6% when *f*_2_ = 1.2 *f*_1_. In Figure 5b, the TVR level was almost zero because the sound waves transmitted from the two cymbal transducers were exactly out of phase, thereby canceling each other.

Comparing the results in Figure 4b and Figure 5a,c, where the notch did not occur, the fractional bandwidths were much larger when the cymbal transducers in the array had the same dielectric polarity direction than when they had opposite polarity directions. Thus, the further design of the broadband cymbal array in this work considered only the case where the dielectric polarity directions of Tx_1_ and Tx_2_ were the same.

## 4. Design of the Wideband 2 × 2 Cymbal Array Pattern

Based on the results presented in Section 3, we designed the structure of the 2 × 2 cymbal array to have the widest fractional bandwidth. The design variables were the center frequency *f*_2_ of Tx_2_ and the CTC spacing. The center frequency of Tx_1_ was fixed to *f*_1_ in the same manner as in Section 3.

**Figure 6:**
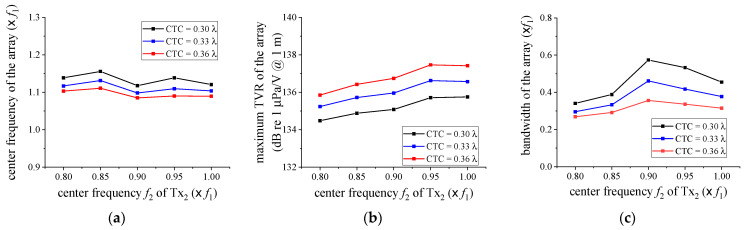
Variations of the acoustic characteristics of the cymbal array in relation to *f*_2_: (**a**) center frequency; (**b**) maximum TVR level; (**c**) bandwidth.

**Figure 7:**
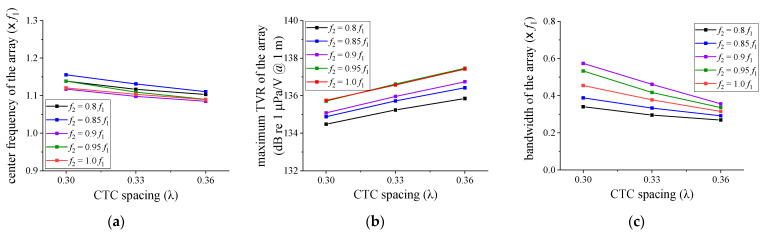
Variations of the acoustic characteristics of the cymbal array in relation to the CTC spacing: (**a**) center frequency; (**b**) maximum TVR level; (**c**) bandwidth.

For a more accurate design of the array structure, the *f*_2_ variation was further subdivided within the range of 0.8 *f*_1_ to 1.0 *f*_1_ by 0.05 *f*_1_ intervals. The variation range of the CTC spacing was also narrowed to the range of 0.30 λ to 0.36 λ. The dimensions of the cymbal transducers with the center frequencies of 0.85 *f*_1_, 0.90 *f*_1_, and 0.95 *f*_1_ were derived in the same manner as that in Section 2 [17]. Figure 6 and Figure 7 summarize the variations of the acoustic characteristics of the cymbal array according to the change of design variables. The extracted performance values were the center frequency, maximum TVR level, and −3 dB bandwidth.

In Figure 6, the center frequency of the array was not significantly affected by the change of *f*_2_. The maximum TVR level of the array increased as *f*_2_ increased for all the CTCs. The array bandwidth showed a maximum at a specific *f*_2_. In Figure 7, the center frequency and the bandwidth of the array decreased as the CTC spacing increased. On the contrary, the maximum TVR level increased, regardless of *f*_2_. These results indicate that the smaller the distance between cymbal transducers, the stronger the interaction between neighboring transducers, which results in an increasing bandwidth, but a decreasing TVR level of the array. The interaction herein refers to the acoustic crosstalk between neighboring transducers in which the sound waves from each transducer are influenced by the interference of its neighboring transducers [17,21]. The results implied that for a wider bandwidth, we had to set *f*_2_ to have a specific ratio to the center frequency *f*_1_ of Tx_1_ and make the CTC spacing smaller. The data on Figure 6 and Figure 7 are representative samples of the analysis results, to illustrate the relationship between the structural parameters (i.e., center frequency *f*_2_ with respect to *f*_1_ and CTC spacing). To achieve the widest array bandwidth, a more general optimal design of the array structure must be made by reflecting on the effects of all the structural parameters.

The effects of the structural parameters in Figure 6 and Figure 7 were not independent, but rather cross-coupled with each other. Hence, a regression analysis was conducted on the data to derive a general relationship between the structural parameters and the array performance, such as the −3 dB fractional bandwidth (FBW_0_) and the maximum TVR level (TVR_c_) of the array within the variation ranges of the parameters [22]. A regression analysis was performed to find a model that best fits the relationship between design variables, by using the least mean square method [23]. Equations (1) and (2) present the FBW_0_ and the TVR_c_, respectively, formulated in terms of design variables *x*_1_ and *x*_2_, where *x*_1_ is the center frequency *f*_2_ as a ratio to *f*_1_, and *x*_2_ is the CTC spacing between the Tx_1_ and Tx_2_ transducers. The effects of these variables are nonlinear; thus, Equations (1) and (2) are expressed as quadratic polynomial functions.

(1) $$\begin{array}{ll} FBW_{0}= & \left(3.0844\times 10\right)+\left(7.3349\times 10^{-1}\right)x_{1}^{2}x_{2}^{2}+4.9171x_{1}^{2}x_{2}^{1}-4.2763x_{1}^{2}\\ & +1.8285x_{1}^{1}x_{2}^{2}+8.3924x_{1}^{1}x_{2}^{1}-6.2635x_{1}^{1}+1.1865x_{2}^{2}-5.6047x_{2}^{1} \end{array}$$

(2) $$\begin{array}{ll} TVR_{C}= & \left(1.3746\times 10^{2}\right)+\left(7.3500\times 10^{-3}\right)x_{1}^{2}x_{2}^{2}-\left(2.3820\times 10^{-2}\right)x_{1}^{2}x_{2}^{1}\\ & +\left(1.9497\times 10^{-1}\right)x_{1}^{2}+\left(2.9850\times 10^{-3}\right)x_{1}^{1}x_{2}^{2}+\left(4.1135\times 10^{-2}\right)x_{1}^{1}x_{2}^{1}\\ & +\left(9.1186\times 10^{-1}\right)x_{1}^{1}-\left(3.1495\times 10^{-2}\right)x_{2}^{2}+\left(8.1128\times 10^{-1}\right)x_{2}^{1} \end{array}$$

This work aimed to design the pattern of a 2 × 2 cymbal array with the widest possible bandwidth. The structure was derived by applying an optimization algorithm (i.e., OptQuest Nonlinear Program (OQNLP) algorithm) to Equations (1) and (2) [24]. The OQNLP algorithm is a multi-start heuristic algorithm specializing in finding global optimizations for pure- and mixed-integer nonlinear problems with many constraints and variables [25]. Equation (3) presents the objective function of the optimization, which is to maximize the array’s fractional bandwidth. As a limiting condition, the maximum TVR level of the array was set higher than or equal to the maximum TVR level of the reference model comprising only Tx_1_ transducers. The optimization process was conducted to determine *f*_2_ and the CTC spacing that could maximize the fractional bandwidth while satisfying the constraint on the TVR level.

Objective: maximize the fractional bandwidth Constraint: TVR ≥ 130.9 dB(3)

**Figure 8:**
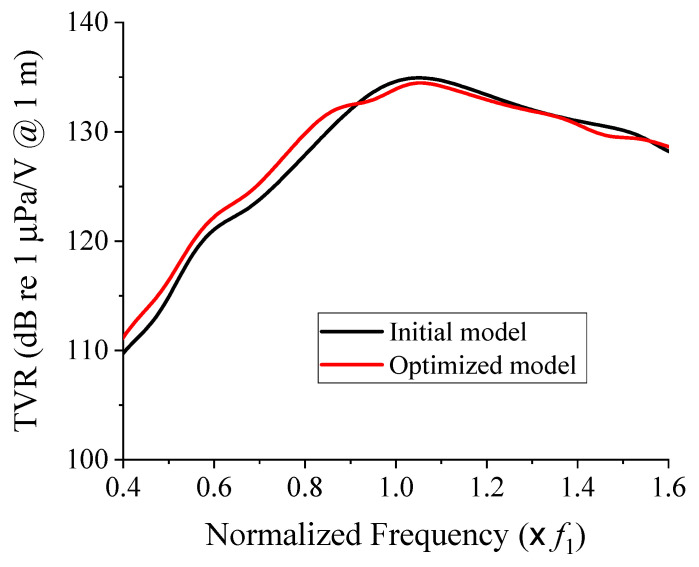
Comparison of the TVR spectra of the initial and optimized cymbal arrays.

The optimization result showed that *f*_2_ should be 0.92 *f*_1_, while the CTC spacing should be 0.30 λ. Figure 8 illustrates the performance of the optimized structure in comparison with that of the initial model. Here, the initial model was a cymbal array composed of Tx_1_ transducers only. Table 3 summarizes a quantitative comparison of the performances. The maximum TVR level of the optimized cymbal array was 134.5 dB, which satisfied the constraint. The fractional bandwidth was increased by 23.7% in comparison with that of the initial model. Even though the same number of transducers was used, the fractional bandwidth could be dramatically increased by optimizing the ratio of the center frequency of Tx_2_ to that of Tx_1_ and the CTC spacing. Meanwhile, the TVR level was maintained above a given requirement, which confirmed the efficacy of the design scheme in this work.

## 5. Fabrication and Characterization of the 2 × 2 Cymbal Array

**Figure 9:**
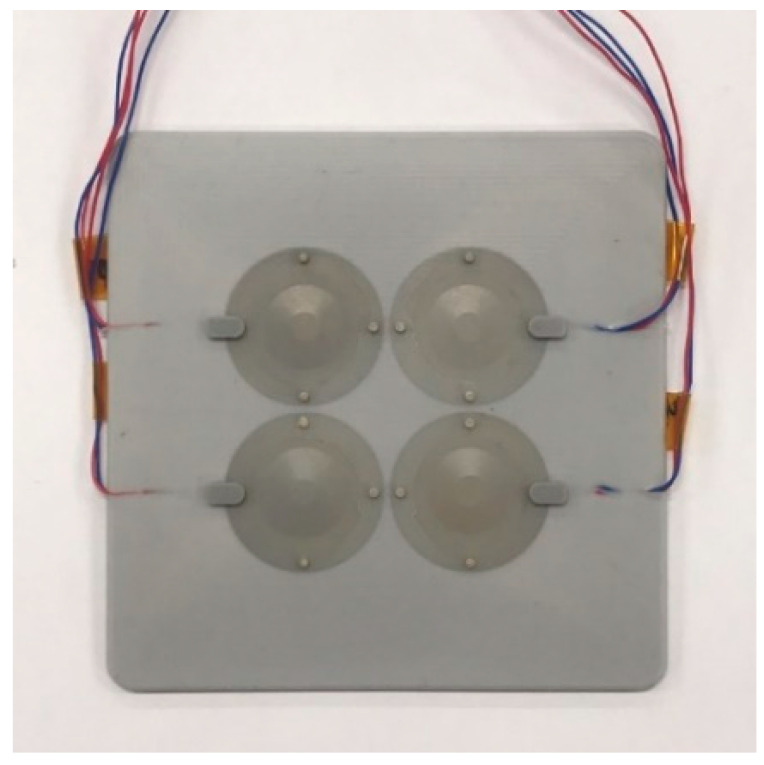
Photograph of the 2 × 2 cymbal array prototype after the second coating.

**Figure 10:**
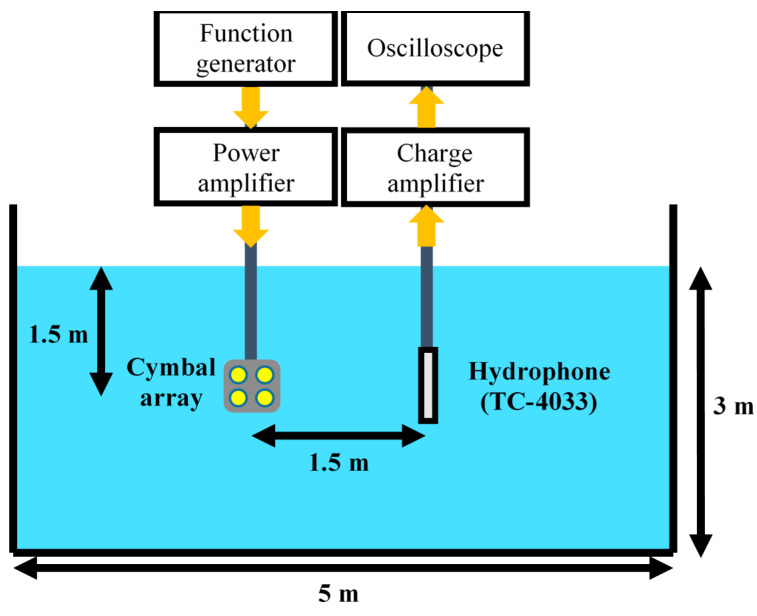
Schematic diagram of the underwater TVR spectrum measurement setup.

To verify the design validity, we fabricated cymbal array prototypes following the design and measured their acoustic characteristics. Both the initial and the optimized models in Section 4 were fabricated to have the same dimensions and materials as those in the FEA models. The metal cap, piezoceramic, and ring were bonded together using epoxy (EB-106, EpoxySet, Inc., RI). The cap and the ring were fastened again with plastic bolts to improve the transducer robustness. The cymbal transducer was coated with RTV-3460 (Elkem, Oslo, Norway) for electrical insulation. This first coating was 0.3 mm thick. After four cymbal transducers were arranged to have the pattern designed in Section 4, the 2 × 2 array was then re-coated with RTV-3460 (Figure 9). The second coating did not cover the metal caps, but filled the gap between the transducers and the edges of the array pattern. The underwater TVR of the cymbal array was measured in the environment illustrated in Figure 10 using the method similar to that in [17].

**Figure 11:**
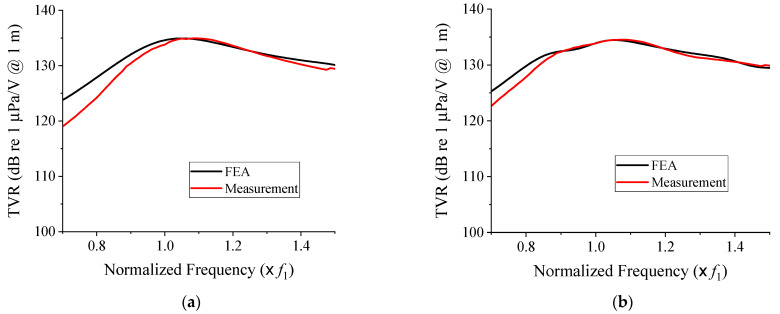
Comparison of the simulated and measured TVR spectra of the prototype cymbal arrays: (**a**) initial; (**b**) optimized models.

Figure 11 shows the measured underwater TVR spectra of the cymbal arrays compared with those from the FEA for both the initial and optimized models. Overall, we found a good agreement between the simulated and measured results. Table 4 summarizes the quantitative comparison of the FEA and the measured data. The center frequencies and the maximum TVR levels from the measurement coincided well with those from the FEA for both the initial and optimized structures. However, the fractional bandwidth showed some discrepancy, which was considered to be partly caused by the tolerance in the fabricated cap dimension. The dimensional tolerance during the mechanical machining process of the caps was in the order of 100 µm. The mechanical tolerance caused some variations in the acoustical characteristics of individual cymbal transducer prototypes, which was responsible for the discrepancy in the array bandwidth performance.

As for the FEA results, the maximum TVR level of the optimized cymbal array satisfied the constraint at 133.7 dB in the measured results. The simulated and measured fractional bandwidths increased by 23.7% and 17.4%, respectively, after the optimization, indicating that the design scheme was effective in widening the bandwidth. This result confirmed the scheme’s efficacy for designing a broadband array pattern of cymbal transducers.

## 6. Conclusions

Cymbal transducers are suitable for constructing a large sensor network because of their structural peculiarity. Previous works on the development and characterization of a cymbal array employed only cymbal transducers with an identical structure and a uniform planar array pattern. This work was the first to conduct a systematic analysis of the effects of the structural parameters of a cymbal array on the array’s acoustic characteristics and to design a broadband array structure by incorporating the effects of the parameters.

The structural parameters considered in this work were the dielectric polarity direction of the piezoceramic disks, the center frequency of each cymbal–transducer pair, and the CTC spacing between the transducers. Using an optimization technique, we derived the ratio of the center frequencies to the CTC spacing between the constituent transducers that could maximize the array bandwidth. The design validity was verified by fabricating and evaluating prototype cymbal arrays and comparing their performances with that of the design. The comparison confirmed the design scheme efficacy for developing a broadband underwater cymbal array.

In this study, we analyzed a 2 × 2 array for the sake of calculation efficiency in the analysis. However, a 2 × 2 array is the simplest form of all the possible array structures and therefore has clear limitations in acoustic power, bandwidth, radiating surface area, and so on, which may restrict the scope of its practical application. As a future work, we will extend the dimension of the array to 3 × 3 and 4 × 4 arrays, and then generalize the design scheme to a cymbal array of an arbitrary dimension.

## Figures and Tables

**Table 1:** Dimensions of the designed Tx_1_ transducer.

Parameter	Symbol	Dimension (mm)
Apex diameter	*d*_a_	5.1
Base diameter	*d*_b_	14.4
Piezoceramic disk diameter	*d*_c_	20.0
Ring width	*W*_r_	3.0
Bond width	*W*_b_	0.3
Cavity height	*h*_c_	0.7
Piezoceramic disk thickness	*t*_c_	1.0
Metal cap thickness	*t*_m_	0.5

**Table 2:** Acoustic characteristics of the designed Tx_1_ and two Tx_2_ transducers.

	Center Frequency	Maximum TVR Level (dB)	Fractional Bandwidth (%)
Tx_1_	*f*_1_	130.9	15.9
Tx_2_ of 0.8 *f*_1_	0.8 *f*_1_	130.7	17.3
Tx_2_ of 1.2 *f*_1_	1.2 *f*_1_	130.9	16.0

**Table 3:** Quantitative comparison of the acoustic characteristics of the initial and optimized cymbal arrays.

	Center Frequency	Maximum TVR (dB)	Fractional Bandwidth (%)
Initial model	1.1 *f*_1_	134.9	37.1
Optimized model	1.1 *f*_1_	134.5	45.9

**Table 4:** Quantitative comparison of the simulated and measured acoustic characteristics of the prototype cymbal arrays: (**a**) initial; (**b**) optimized models.

(**a**)			
	**Center Frequency**	**Maximum TVR (dB)**	**Fractional Bandwidth (%)**
FEA	1.1 *f*_1_	134.9	37.1
Measurement	1.1 *f*_1_	134.9	31.7
(**b**)			
	**Center Frequency**	**Maximum TVR (dB)**	**Fractional Bandwidth (%)**
FEA	1.1 *f*_1_	134.5	45.9
Measurement	1.1 *f*_1_	133.7	37.2

## Data Availability

Not applicable.
